# Zika virus outbreak: reproductive effects and decreases in the number of births in Brazil

**DOI:** 10.5935/1518-0557.20170064

**Published:** 2017

**Authors:** Paulo F Taitson, Vanessa GM de Souza, Mariana L Santos

**Affiliations:** 1 Unit of Human Reproduction, DCB/Pontifical Catholic University of Minas Gerais, Brazil

An infectious agent new to Brazil arrived in our country in 2015, to cause significant social impact and uproar in the scientific community in the areas of human health and reproduction. The Zika virus, detected in past decades in Africa and Southeast Asia, first appeared in the Brazilian Northeast in October of the said year, primarily in the State of Pernambuco, where 25.9% of the suspected cases were observed. The outbreak stretched into the following year, causing civil unrest, the issuance of health alerts, and apprehension in the scientific community as more information and studies on the infectious disease were needed (Carvalho *et al*., 2016).

The initial symptoms of infection are low-grade fever, maculopapular rash, conjunctivitis, and arthralgia, accompanied by central nervous system disorders caused by the onset microcephaly in intrauterine life. The latter raised questions around the medium and long-term impacts on the motor and cognitive development of affected children, and prompted the establishment of preventive measures and research on other possible virus transmission pathways. As in the case of dengue, a disease already present in Brazil, the Zika virus is also transmitted by a mosquito of the Aedes genus (*Aedes aegypti* vs. *Aedes albopictus*). But the Zika virus is not transmitted solely through a simple mosquito bite. Other pathways include blood transfusion, sexual intercourse, and transplacental transmission. Additional possibilities are being studied (Baud *et al*., 2017).

Transmission through sexual intercourse has stimulated discussions on the possible effects it might have in the field of human reproduction. Studies are being held to confirm the time for which the infectious agent stays in the human body. Dissemination of the virus has been reported to occur 41 days after the onset of initial symptoms, but the maximum length of the contagious period has not been established yet. To further complicate matters, infective viral particles have been detected in semen 69 days after infection, while viral RNA has been detected through reverse-transcription (RT)-PCR as many as 188 days after infection (Baud *et al*., 2017). 

Despite the lack of studies devised to gather more evidence on the presence and behavior of the virus in gametes and in the reproductive apparatus of individuals of both sexes, semen infected with the Zika virus has been the topic of several study submissions looking into pathogen-gamete interactions and the possibility of infectious materials making it to human oocytes. In published studies, gametes and animal embryos were susceptible to infection by viral antigens despite glycoprotein-based protection mechanisms such as the zona pellucida, although long-term developmental implications have not been analyzed. These findings may serve as the foundation for the risk factors connected to the transmission of Zika virus in assisted reproductive technologies, since follicular fluid may carry the Zika virus (Washington *et al*., 2016). 

Although the site of viral replication in humans has not been determined, it is believed to be located in the prostate or seminal vesicles, preferentially in the testicular and epididymal region, potentially extending the duration of the sexual transmission potential. Through analogies with other studies on brain tissue, the Zika virus is believed to preferentially infect progenitor cells, making spermatogonia its main target. The appearance of orchitis and epididymitis in mice up to sixty days after infection demonstrated the potential of the pathogen to cause tissue damage in the long-term by innate immune response. Evidence of interference with cell division in the testicles poses another significant challenge to male fertility, since it may induce the death of spermatozoa, infertility, and potentially azoospermia, in addition to possible effects on mature sperm cells such as decreasing their motility and ability to fertilize oocytes. The female genitourinary tract has also been analyzed as a possible reservoir of ZIKV and a potential source of chronic risk to fetuses, two factors that significantly complicate prenatal counseling. Nonetheless, the role of bodily fluids and the urinary systems of both sexes in the extension of the infectious potential of viral RNA needs to be better investigated and clarified (Baud *et al*., 2017; Govero *et al*., 2016).

Fear of the complications associated with infection by the Zika virus have led to a reduction in the number of births in the State of Pernambuco. Hospitals once overcrowded started to have empty beds by the end of 2016. In September of 2015, before the issuance of Zika virus warnings, some 8,000 births were recorded in the State of Pernambuco. In November of 2016, just over ten months since the declaration of state of emergency on account of cases of microcephaly, the number of births dropped by an astounding 27%. In Campinas, São Paulo, the number of births decreased by more than 5.0% in 2016 in relation to 2015, breaking a decade-long string of annual growth rates of two percent. Two reasons were listed by the local Secretary of Health to explain the decrease in the number of births: the economic crisis the country is going through, which may have forced couples to postpone their plans of having children, and the cases of Zika virus infection, which significantly increased the number of babies born with microcephaly. In Belo Horizonte, Minas Gerais, many maternity hospitals recorded decreases in the number of births in the first trimester of 2017. The State's Capital also had cases of Zika virus infection. The combination of generalized fear of an outbreak and awareness of the disease's complications pulled the number of births down by more than 20%, particularly among women aged 20-30 years. 
